# STAT1 is essential for the inhibition of hepatitis C virus replication by interferon-λ but not by interferon-α

**DOI:** 10.1038/srep38336

**Published:** 2016-12-08

**Authors:** Shota Yamauchi, Kenji Takeuchi, Kazuyasu Chihara, Chisato Honjoh, Yuji Kato, Hatsumi Yoshiki, Hak Hotta, Kiyonao Sada

**Affiliations:** 1 Department of Genome Science and Microbiology, Faculty of Medical Sciences, University of Fukui, Fukui 910-1193, Japan; 2 Life Science Innovation Center, University of Fukui, Fukui 910-1193, Japan; 3 Third Department of Internal Medicine, Faculty of Medical Sciences, University of Fukui, Fukui 910-1193, Japan; 4 Department of Otorhinolaryngology Head and Neck Surgery, Faculty of Medical Sciences, University of Fukui, Fukui 910-1193, Japan; 5 Division of Microbiology, Kobe University Graduate School of Medicine, Kobe 650-0017, Japan

## Abstract

Interferon-α (IFN-α) and IFN-λ are structurally distinct cytokines that bind to different receptors, but induce expression of similar sets of genes through Janus kinase (JAK)-signal transducers and activators of transcription (STAT) pathways. The difference between IFN-α and IFN-λ signaling remains poorly understood. Here, using the CRISPR/Cas9 system, we examine the role of STAT1 and STAT2 in the inhibition of hepatitis C virus (HCV) replication by IFN-α and IFN-λ. Treatment with IFN-α increases expression of IFN-stimulated genes (ISGs) such as double-stranded RNA-activated protein kinase (PKR) and decreases viral RNA and protein levels in HCV-infected Huh-7.5 human hepatoma cells. These responses are only partially attenuated by knockout of STAT1 but are abolished by knockout of STAT2. In contrast, the inhibition of HCV replication by IFN-λ is abolished by knockout of STAT1 or STAT2. Microarray analysis reveals that IFN-α but not IFN-λ can induce expression of the majority of ISGs in STAT1 knockout cells. These findings suggest that IFN-α can inhibit HCV replication through a STAT2-dependent but STAT1-independent pathway, whereas IFN-λ induces ISG expression and inhibits HCV replication exclusively through a STAT1- and STAT2-dependent pathway.

Interferon-α (IFN-α) and IFN-β (also called type I IFNs and hereafter IFN-α/β) are antiviral cytokines that signal through the IFN-α receptor (IFNAR)^1^. Upon binding to IFN-α/β, IFNAR activates the receptor-associated tyrosine kinases Janus kinase 1 (JAK1) and tyrosine kinase 2, which in turn phosphorylate signal transducers and activators of transcription 1 (STAT1) and STAT2. Phosphorylated STAT1 and STAT2 heterodimerize and associate with IFN-regulatory factor 9 (IRF9) to form a transcription factor complex known as IFN-stimulated gene factor 3 (ISGF3). ISGF3 translocates to the nucleus, where it binds to IFN-stimulated response elements (ISREs) within the promoters of hundreds of IFN-stimulated genes (ISGs), thereby activating their transcription. Some ISG products have been established as antiviral effectors^2^. For example, double-stranded RNA-activated protein kinase (PKR) inhibits viral and cellular protein synthesis by phosphorylating the α subunit of eukaryotic initiation factor 2 (ref. 3). In addition to antiviral effectors, the components of ISGF3 are themselves encoded by ISGs^2^.

STAT2 was discovered as a component of ISGF3 (ref. 4). However, accumulating evidence indicates that STAT2 can mediate IFN-α-induced ISG expression independently of STAT1, at least in certain cell types^5^. For example, recent studies have shown that knockout of STAT2, but not STAT1, abolishes IFN-α-induced expression of ISG15 and myxovirus resistance 1 (MX1) in bone marrow-derived macrophages and adenosine deaminase acting on RNA 1 in mouse embryonic fibroblasts^6^,^7^. In addition to forming ISGF3, STAT2 can homodimerize and associate with IRF9 to form an ISGF3-like complex^5^,^8^. This complex not only substitutes for ISGF3 but also has its unique target genes^9^. STAT2 can also heterodimerize with STAT3 or STAT6 (ref. 5). It has been speculated that STAT1-independent IFN-α signaling may have evolved to counter viruses that attempt to evade host immune responses by targeting STAT1 (e.g., Sendai virus^10^)^5^.

IFN-λ, also known as type III IFN, was first reported as an antiviral cytokine similar to IFN-α/β in 2003 (refs 11,12). Like IFN-α/β, IFN-λ induces ISG expression through the formation of ISGF3 (refs 13, 14, 15). However, IFN-λ is structurally more similar to members of the IL-10 family than IFN-α/β^16^. Consistent with this structural similarity, the IFN-λ receptor (IFNLR) consists of the unique IFN-λ receptor chain 1 and the shared IL-10 receptor chain 2 (refs 13,14). Unlike IFNAR, which is expressed on almost all cell types, IFNLR is expressed primarily on mucosal epithelial cells and hepatocytes. Notably, intestinal epithelial cells respond preferentially to IFN-λ over IFN-α/β^17^. IFN-λ is therefore essential for the control of intestinal rotavirus and norovirus infection *in vivo*^17^,^18^.

Hepatocytes respond to both IFN-α/β and IFN-λ^13^,^14^. IFN-α and IFN-λ induce phosphorylation of STAT1 and STAT2 and increase expression of similar sets of ISGs in hepatocytes, but with distinct kinetics^19^,^20^. Furthermore, although both IFN-α and IFN-λ have antiviral activity against viruses such as hepatitis C virus (HCV), hepatitis B virus, encephalomyocarditis virus, and herpes simplex virus type 2 (HSV-2), IFN-α is markedly more potent against HSV-2 than IFN-λ^19^,^21^,^22^,^23^. The reason for these differences between IFN-α and IFN-λ in hepatocytes is unknown. However, recent studies have indicated that unlike IFN-α/β, IFN-λ induces phosphorylation of JAK2, raising the possibility that IFN-α/β and IFN-λ activate different JAK-STAT pathways^24^,^25^.

In this study, using the CRISPR/Cas9 system, we show that IFN-α can inhibit HCV replication independently of STAT1 in Huh-7.5 human hepatoma cells. We also show that unlike IFN-α, IFN-λ induces expression of the majority of ISGs and inhibits HCV replication exclusively through a STAT1-dependent pathway. Our results suggest that IFN-α and IFN-λ signaling differ in their dependence on STAT1.

## Results

### STAT1 is not essential for the inhibition of HCV replication by IFN-α

**Figure 1:**
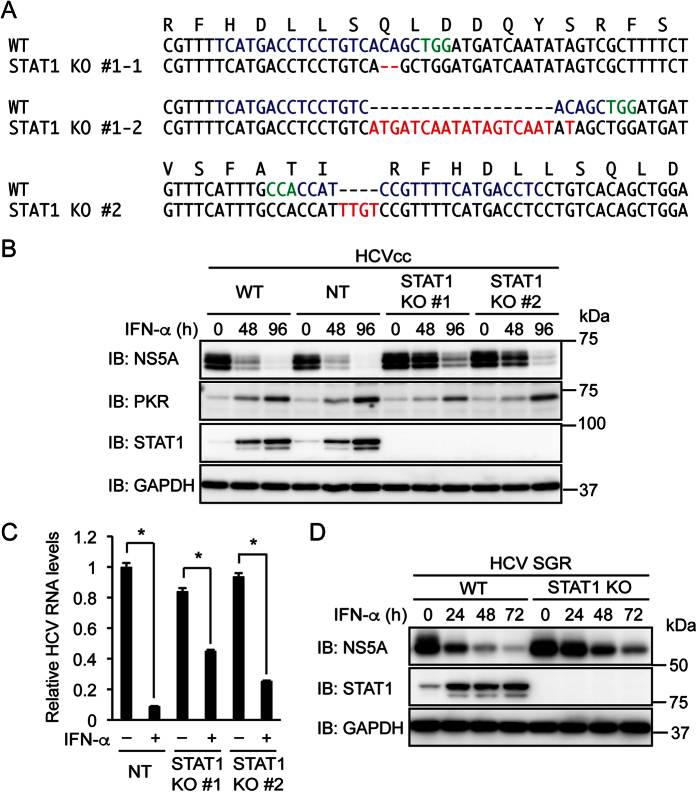
Knockout of STAT1 attenuates but does not abolish the inhibition of HCV replication by IFN-α. (**A**) STAT1 knockout (KO) clones #1 and #2 were established from Huh-7.5 cells expressing STAT1 sgRNAs #1 and #2, respectively. DNA and amino acid sequences surrounding the sgRNA target sequences (blue) are shown. The protospacer adjacent motif (PAM) and the mutation in each allele are shown in green and red, respectively. (**B**) WT cells, NT sgRNA-expressing cells, and STAT1 KO cells (clones #1 and #2) were infected with HCVcc and treated with IFN-α (1,000 U/ml) for the indicated times. The expression levels of NS5A, PKR, and STAT1 were evaluated by immunoblotting (IB). (**C**) NT sgRNA-expressing cells and STAT1 KO cells were infected with HCVcc and treated with IFN-α (1,000 U/ml) for 96 h. Intracellular HCV RNA levels were quantified by real-time PCR and normalized to control values. Data represent the mean ± S.D. (*n* = 3). *P < 0.01. (**D**) WT and STAT1 KO (STAT1 sgRNA #2) Huh-7.5 cells harboring HCV subgenomic replicon (SGR) were treated with IFN-α (1,000 U/ml) for the indicated times. The expression levels of NS5A and STAT1 were evaluated by immunoblotting.

IFN-α is thought to induce ISG expression primarily through the formation of ISGF3, a transcription factor complex composed of STAT1, STAT2, and IRF9 (ref. 1). To determine whether the inhibition of HCV replication by IFN-α requires STAT1, two clones of STAT1 knockout Huh-7.5 human hepatoma cells were established using the CRISPR/Cas9 system. A frame shift mutation or a nonsense mutation was introduced into each allele of STAT1 (Fig. 1A). A clone of Huh-7.5 cells expressing a non-targeting (NT) sgRNA was also established to use as control cells. Wild-type (WT) Huh-7.5 cells, NT sgRNA-expressing cells, and STAT1 knockout cells were infected with cell culture-grown HCV (HCVcc) of the J6/JFH1 chimeric genome and treated with IFN-α. As expected, IFN-α treatment decreased the HCV nonstructural protein 5 A (NS5A) in WT cells and NT sgRNA-expressing cells (Fig. 1B). This decrease was partially attenuated but not abolished in STAT1 knockout cells. IFN-α treatment increased PKR expression in WT cells and NT sgRNA-expressing cells, and this increase was also not abolished in STAT1 knockout cells. Consistent with the decrease in NS5A expression, IFN-α treatment decreased HCV RNA in both control cells and STAT1 knockout cells (Fig. 1C). Similar results regarding NS5A were obtained with Huh-7.5 cells harboring HCV subgenomic replicon (Fig. 1D). In contrast, IFN-γ treatment decreased NS5A in NT sgRNA-expressing cells but not in STAT1 knockout cells (Supplementary Fig. S1). This is consistent with the central role of STAT1 in IFN-γ signaling^1^. These results suggest that STAT1 is involved in but not essential for the inhibition of HCV replication by IFN-α.

### STAT2 and IRF9 are essential for the inhibition of HCV replication by IFN-α

**Figure 2:**
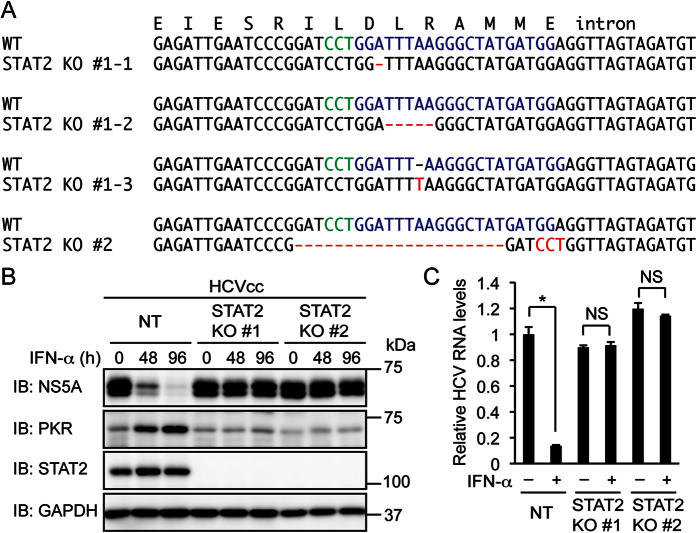
Knockout of STAT2 abolishes the inhibition of HCV replication by IFN-α. (**A**) STAT2 knockout (KO) clones #1 and #2 were established from Huh-7.5 cells expressing a STAT2 sgRNA. DNA and amino acid sequences surrounding the sgRNA target sequences (blue) are shown. The protospacer adjacent motif (PAM) and the mutation in each allele are shown in green and red, respectively. (**B**) NT sgRNA-expressing cells and STAT2 KO cells (clones #1 and #2) were infected with HCVcc and treated with IFN-α (1,000 U/ml) for the indicated times. The expression levels of NS5A, PKR, and STAT2 were evaluated by immunoblotting (IB). (**C**) NT sgRNA-expressing cells and STAT2 KO cells were infected with HCVcc and treated with IFN-α (1,000 U/ml) for 96 h. Intracellular HCV RNA levels were quantified by real-time PCR and normalized to control values. Data represent the mean ± S.D. (*n* = 3). *P < 0.01. NS, not significant.

Accumulating evidence indicates that a STAT2 complex lacking STAT1 can substitute for ISGF3 in IFN-α signaling^5^. To determine whether STAT2 is required for the inhibition of HCV replication by IFN-α, STAT2 knockout Huh-7.5 cells were established. A frame shift mutation or a mutation probably disrupting a splicing signal^26^ was introduced into each allele of STAT2 (Fig. 2A). IFN-α treatment did not decrease NS5A or viral RNA and did not increase PKR expression in HCVcc-infected STAT2 knockout cells (Fig. 2B and C). These results suggest that STAT2 is essential for the inhibition of HCV replication by IFN-α.

**Figure 3:**
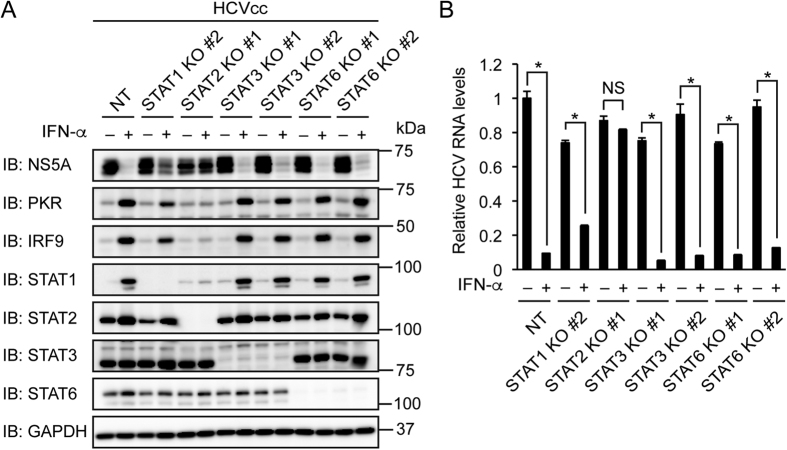
Knockout of STAT3 or STAT6 does not affect the inhibition of HCV replication by IFN-α. (**A**,**B**) STAT3 knockout (KO) clones #1, #2 were established from Huh-7.5 cells expressing STAT3 sgRNAs #1 and #2, respectively. STAT6 knockout (KO) clones #1 and #2 were established from Huh-7.5 cells expressing STAT6 sgRNAs #1 and #2, respectively. (**A**) Cells were infected with HCVcc and treated with IFN-α (1,000 U/ml) for 72 h. The expression levels of NS5A, PKR, IRF9, STAT1, STAT2, STAT3, and STAT6 were evaluated by immunoblotting (IB). (**B**) Cells were infected with HCVcc and treated with IFN-α (1,000 U/ml) for 96 h. Intracellular HCV RNA levels were quantified by real-time PCR and normalized to control values. Data represent the mean ± S.D. (*n* = 3). *P < 0.01. NS, not significant.

STAT2 can heterodimerize with STAT3 or STAT6 (ref. 5). We investigated the possibility that STAT3 or STAT6 is involved in the inhibition of HCV replication by IFN-α. IFN-α treatment decreased NS5A and viral RNA and increased PKR, IRF9, STAT1, and STAT2 in STAT3 knockout cells and STAT6 knockout cells (Fig. 3A and B). These results suggest that neither STAT3 nor STAT6 is involved in the inhibition of HCV replication by IFN-α.

**Figure 4:**
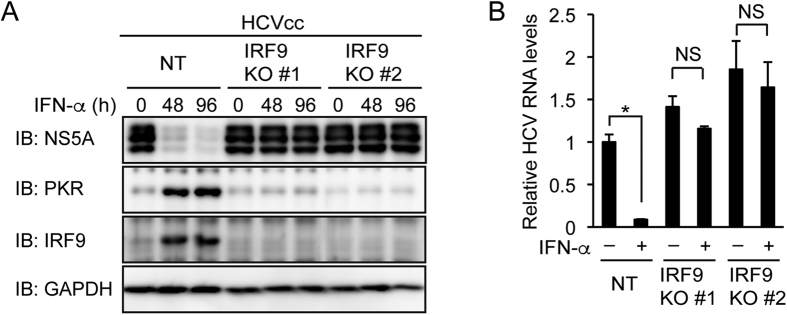
Knockout of IRF9 abolishes the inhibition of HCV replication by IFN-α. (**A**,**B**) IRF9 knockout (KO) clones #1 and #2 were established from Huh-7.5 cells expressing IRF9 sgRNAs #1 and #2, respectively. (**A**) NT sgRNA-expressing cells and IRF9 KO cells (clones #1 and #2) were infected with HCVcc and treated with IFN-α (1,000 U/ml) for the indicated times. The expression levels of NS5A, PKR, and IRF9 were evaluated by immunoblotting (IB). (**B**) NT sgRNA-expressing cells and IRF9 KO cells were infected with HCVcc and treated with IFN-α (1,000 U/ml) for 96 h. Intracellular HCV RNA levels were quantified by real-time PCR and normalized to control values. Data represent the mean ± S.D. (*n* = 3). *P < 0.01. NS, not significant.

STAT2 can homodimerize and associate with IRF9 (ref. 5). We established IRF9 knockout Huh-7.5 cells. IFN-α treatment did not significantly decrease NS5A or viral RNA and did not increase PKR in IRF9 knockout cells (Fig. 4A and B). These results suggest that IRF9 is essential for the inhibition of HCV replication by IFN-α.

### STAT1 and STAT2 are essential for the inhibition of HCV replication by IFN-λ

**Figure 5:**
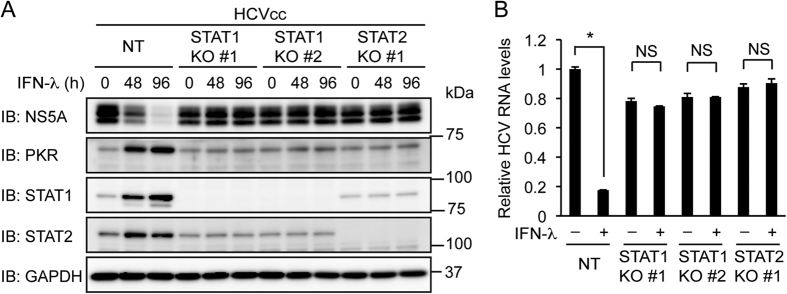
Knockout of STAT1 or STAT2 abolishes the inhibition of HCV replication by IFN-λ. (**A**) NT sgRNA-expressing Huh-7.5 cells, STAT1 knockout (KO) cells (clones #1 and #2), and STAT2 KO cells (clone #1) were infected with HCVcc and treated with IFN-λ (1,000 U/ml) for the indicated times. The expression levels of NS5A, PKR, STAT1, and STAT2 were evaluated by immunoblotting (IB). (**B**) NT sgRNA-expressing cells, STAT1 KO cells (clones #1 and #2), and STAT2 KO cells (clone #1) were infected with HCVcc and treated with IFN-λ (1,000 U/ml) for 96 h. Intracellular HCV RNA levels were quantified by real-time PCR and normalized to control values. Data represent the mean ± S.D. (*n* = 3). *P < 0.01. NS, not significant.

Like IFN-α, IFN-λ is thought to induce ISG expression primarily through the formation of ISGF3 (refs 13,14). To determine whether STAT1 and STAT2 are required for the inhibition of HCV replication by IFN-λ, control Huh-7.5 cells, STAT1 knockout cells, and STAT2 knockout cells were infected with HCVcc and treated with IFN-λ. Treatment with IFN-λ decreased NS5A and viral RNA and increased PKR, STAT1, and STAT2 in control cells (Fig. 5A and B). However, in contrast to treatment with IFN-α, treatment with IFN-λ did not decrease NS5A or viral RNA and did not increase PKR or STAT2 in STAT1 knockout cells. Similar results were obtained with STAT2 knockout cells (Fig. 5A and B), but not with STAT3 or STAT6 knockout cells (Supplementary Fig. S2). These results suggest that the inhibition of HCV replication by IFN-λ requires both STAT1 and STAT2.

### STAT1 plays an essential role in ISG induction by IFN-λ but not by IFN-α

**Figure 6:**
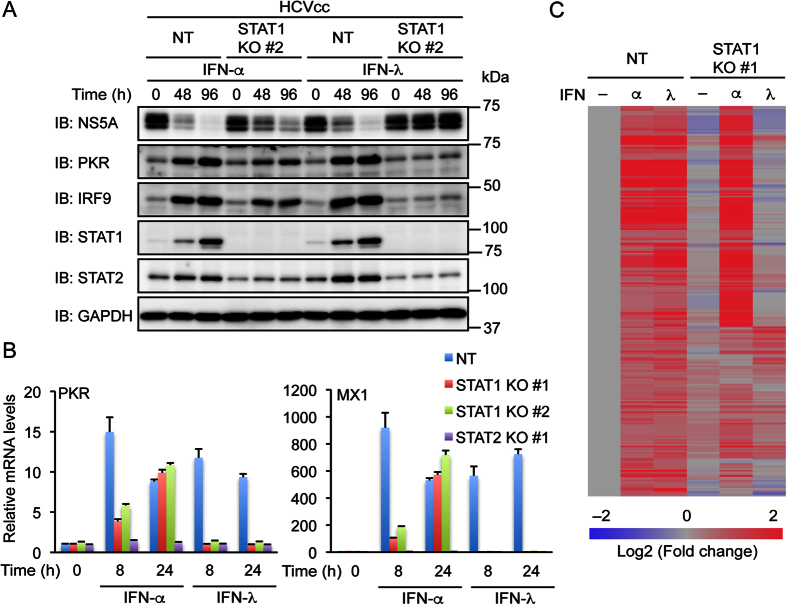
Knockout of STAT1 abolishes induction of ISGs by IFN-λ but not by IFN-α. (**A**) NT sgRNA-expressing Huh-7.5 cells and STAT1 knockout (KO) cells (clone #2) were infected with HCVcc and treated with IFN-α (1,000 U/ml) or IFN-λ (1,000 U/ml) for the indicated times. The expression levels of NS5A, PKR, IRF9, STAT1, and STAT2 were evaluated by immunoblotting (IB). (**B**) NT sgRNA-expressing cells, STAT1 KO cells (clones #1 and #2), and STAT2 KO cells (clone #1) were treated with IFN-α (1,000 U/ml) or IFN-λ (1,000 U/ml) for the indicated times. The levels of PKR and MX1 mRNAs were evaluated by real-time PCR and normalized to control values. Data represent the mean ± S.D. (*n* = 3). (**C**) NT sgRNA-expressing cells and STAT1 KO cells (clone #1) were treated with IFN-α (1,000 U/ml) or IFN-λ (1,000 U/ml) for 24 h. Microarray analysis was performed. Fold changes relative to untreated NT sgRNA-expressing cells were calculated. Probe sets that showed >1.5-fold increase in response to both IFN-α and IFN-λ in NT sgRNA-expressing cells but showed little changes (within 1.5-fold) due to STAT1 KO were selected. Heat maps were generated using the microarray data. See Supplementary Dataset 1 for a full list of the selected probe sets and fold changes.

We compared the effects of IFN-α and IFN-λ on ISG expression in control Huh-7.5 cells and STAT1 knockout cells. Immunoblotting analysis showed that treatment with IFN-λ increased PKR, IRF9, STAT1, and STAT2 similarly to treatment with IFN-α in HCVcc-infected control cells (Fig. 6A). Nevertheless, only IFN-α treatment increased these proteins (except STAT1) to some extent in STAT1 knockout cells.

We also examined mRNA levels of PKR (encoded by *EIF2AK2*) and MX1 by real-time PCR. Treatment with IFN-α or IFN-λ for 8 h or 24 h increased mRNAs of PKR and MX1 in uninfected control cells (Fig. 6B). In contrast, treatment with IFN-α but not IFN-λ increased these mRNAs in STAT1 knockout cells, suggesting that STAT1 is not essential for the induction of PKR or MX1 by IFN-α. However, it should be noted that treatment with IFN-α for 8 h increased PKR and MX1 mRNAs more robustly in control cells than in STAT1 knockout cells. This result suggests that STAT1 is involved in the early induction of these ISGs in response to IFN-α and is consistent with a recent study using primary macrophages^6^. PKR and MX1 mRNA levels were largely unaffected by treatment with IFN-α or IFN-λ in STAT2 knockout cells.

The ISGs that are involved in the inhibition of HCV replication have not been fully identified^27^. To extend the finding that STAT1 is essential for the induction of specific ISGs by IFN-λ but not by IFN-α, microarray analysis was performed using control Huh-7.5 cells and STAT1 knockout cells. The genes that were up-regulated by both IFN-α and IFN-λ in control cells were analyzed. Many of these genes, including well-known ISGs such as IFI6 and IFIT1, were also up-regulated by 24 h treatment with IFN-α in STAT1 knockout cells (Fig. 6C and Table 1). Some ISGs such as SAMD9 and HERC5 showed higher expression in response to IFN-α in STAT1 knockout cells than in control cells, whereas others such as LGALS3BP and BST2 showed the opposite expression pattern. In contrast to IFN-α, IFN-λ increased expression of only a limited number of genes (such as FCN2) in STAT1 knockout cells (see Supplementary Dataset 1). These results suggest that STAT1 is essential for induction of the majority of ISGs by IFN-λ but not by IFN-α.

**Figure 7:**
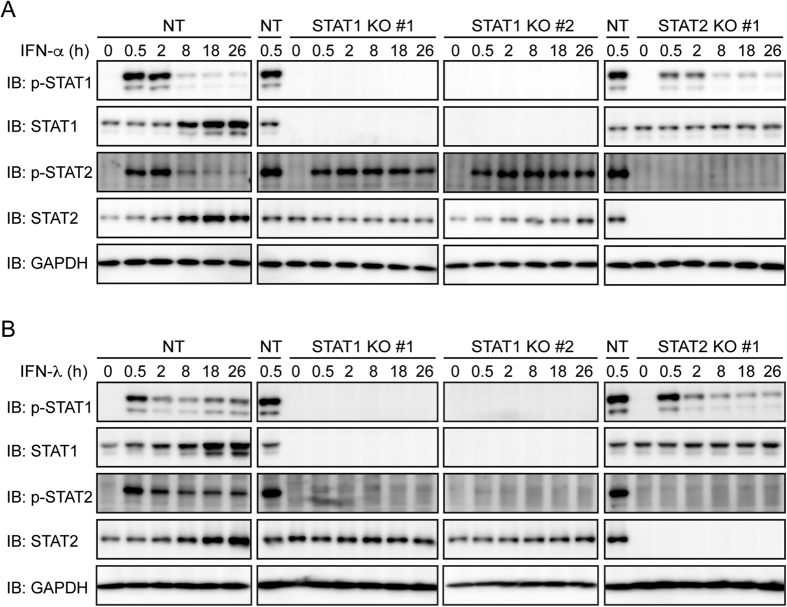
Knockout of STAT1 abolishes IFN-λ-induced phosphorylation of STAT2. (**A**,**B**) NT sgRNA-expressing Huh-7.5 cells, STAT1 knockout (KO) cells (clones #1 and #2), and STAT2 KO cells (clone #1) were treated with IFN-α (1,000 U/ml) (**A**) or IFN-λ (1,000 U/ml) (**B**) for the indicated times. Phosphorylation of STAT1 and STAT2 was evaluated by immunoblotting (IB).

Phosphorylation of STAT1 and STAT2 is required for ISGF3 formation^1^. Phosphorylated STAT2 can also homodimerize and associate with IRF9 to form an ISGF3-like complex^5^. Treatment with IFN-α or IFN-λ induced phosphorylation of STAT1 and STAT2 in control Huh-7.5 cells (Fig. 7A and B). IFN-α-induced phosphorylation of STAT2 was prolonged in STAT1 knockout cells (Fig. 7A) as previously reported^6^. In contrast, IFN-λ-induced phosphorylation of STAT2 was abolished in STAT1 knockout cells (Fig. 7B). These results suggest that STAT1 is essential for STAT2 phosphorylation induced by IFN-λ but not by IFN-α.

## Discussion

**Figure 8:**
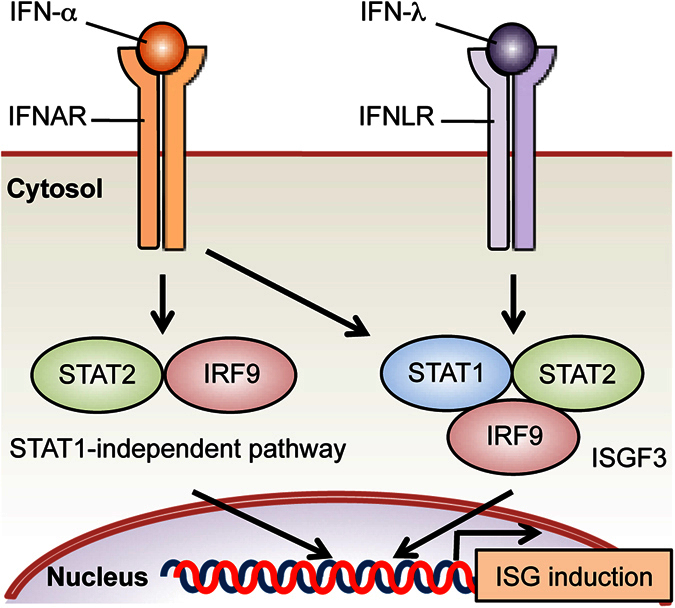
Schematic of the difference between IFN-α and IFN-λ signaling.

IFN-λ is the most recently discovered member of the IFN family. The difference between IFN-α/β and IFN-λ has been intensively studied. Consistent with previous studies^5^,^6^, our results using Huh-7.5 hepatoma cells suggest that both STAT1 and STAT2 are involved in the early induction of ISGs in response to IFN-α, most likely through the formation of ISGF3, but that when IFN-α stimulation is prolonged, ISGs can be induced by a STAT2-dependent, STAT1-independent pathway (Fig. 8). Accordingly, IFN-α can inhibit HCV replication to some extent in the absence of STAT1 (Fig. 1). This inhibition requires STAT2 (Fig. 2) and IRF9 (Fig 4) but does not appear to require STAT3 or STAT6 (Fig. 3). Furthermore, IFN-α can induce STAT2 phosphorylation in STAT1 knockout cells (Fig. 7). Previous studies have shown that expression of a fusion protein of STAT2 and IRF9 inhibits the replication of viruses including HCV in the absence of STAT1 (refs 28, 29). It is therefore likely that IFN-α can inhibit HCV replication through the STAT2/IRF9 complex^9^. Our results also suggest that unlike IFN-α, IFN-λ induces expression of the majority of ISGs and inhibits HCV replication exclusively through a STAT1-dependent pathway. This difference between IFN-α and IFN-λ signaling may possibly explain the fact that the genes induced by IFN-λ are typically a subset of the genes induced by IFN-α/β^13^,^15^,^30^. Our microarray analysis indicated that IFN-α induced expression of ISGs such as OAS2 and IFIT1 in STAT1 knockout Huh-7.5 cells (Table 1). In contrast, the induction of these ISGs is abrogated in STAT1 knockout fibrosarcoma cells^9^. Interestingly, overexpression of STAT2 in these cells restores the induction of OAS2 and IFIT1 and also enables induction of genes that do not contain ISREs. Endogenous STAT2 may also mediate ISRE-independent gene induction in response to IFN-α, at least in certain cell types.

Only ~20% of people infected with HCV spontaneously clear the virus, in part because HCV evades host immune responses by various mechanisms^31^. For example, HCV decreases RIG-I-like receptor-dependent IFN production by cleaving mitochondrial antiviral signaling protein (MAVS) in infected cells^32^,^33^. HCV has also been proposed to suppress ISGF3-mediated gene induction^31^. When overexpressed, the HCV core protein binds to STAT1 and prevents its phosphorylation^34^. However, a study using a luciferase reporter assay showed that HCVcc infection does not block ISRE-dependent gene induction in response to IFN-β^35^. This may suggest that like IFN-α, IFN-β can induce ISG expression independently of STAT1. Indeed, treatment with INF-β decreased NS5A in STAT1 knockout cells (Supplementary Fig. S1). We showed that knockout of STAT1 abolished the induction of ISGs such as PKR and IRF9 by IFN-λ in HCVcc-infected cells (Fig. 5A), suggesting that the inhibition of STAT1-dependent IFN-λ signaling by HCVcc is incomplete. It would be interesting to determine whether IFN-α has more potent antiviral activity than IFN-λ against other viruses that are thought to inhibit STAT1.

HCV is a hepatotropic RNA virus that causes liver cirrhosis and hepatocellular carcinoma^36^. Until recently, recombinant IFN-α, alone or in combination with ribavirin, was the only therapeutic option for patients infected with HCV. IFN-α is also produced by plasmacytoid dendritic cells that have received viral RNA from HCV-infected hepatocytes via exosomes^37^. HCV-infected hepatocytes, on the other hand, preferentially produce IFN-λ^38^. Humans have four IFN-λ genes (*IFNL1, IFNL2, IFNL3*, and *IFNL4*)^39^. IFN-λ1 was used throughout this study. We have confirmed that IFN-λ2 treatment is also unable to decrease NS5A in STAT1 knockout cells (Supplementary Fig. S1). The newly discovered IFN-λ4 is expressed only in people who carry the *IFNL4*-ΔG allele and not in the others because of a frame shift mutation. When infected with HCV, people carrying the *IFNL4*-ΔG allele show higher ISG expression and lower HCV RNA levels in the liver. This is consistent with the antiviral activity of IFN-λ4 observed in cell culture^40^. However, people carrying the *IFNL4*-ΔG allele are less likely to clear HCV infection spontaneously or in response to IFN-α-based therapy. The reason for this paradox is unknown, but IFN-λ4 has been speculated to render HCV-infected hepatocytes refractory to IFN-α, for example by inducing the expression of USP18, which inhibits IFN-α but not IFN-λ signaling^41^. It is tempting to speculate that hepatocyte-produced IFN-λ4 is incapable of clearing HCV infection *in vivo*, at least in part because of its inability to induce STAT1-independent gene expression.

## Methods

### Cell culture and HCV infection

Huh-7.5 human hepatoma cells were kindly provided by Charles M. Rice (The Rockefeller University) and were cultured in DMEM (Wako) supplemented with 10% FBS (Sigma-Aldrich) and 0.1 mM nonessential amino acids (Wako). Huh-7.5 cells harboring HCV subgenomic replicon (Con1) were described previously^42^,^43^. HEK293T cells were cultured in DMEM supplemented with 10% FBS. HCV (J6/JFH1) infection was performed as described previously^44^,^45^. Infected cells were incubated for 48 h prior to experiments using IFNs.

### Antibodies and reagents

Anti-STAT1 (sc-464), anti-STAT2 (sc-476), anti-STAT3 (sc-7179), anti-PKR (sc-707), anti-NS5A (sc-52417), and anti-IRF9 (sc-496) antibodies were purchased from Santa Cruz Biotechnology. Anti-GAPDH (#MAB374) antibody was purchased from Merck Millipore. Anti-STAT6 (#5397) and anti-phospho-STAT1 (#7649) antibodies were purchased from Cell Signaling Technology. Anti-phospho-STAT2 (600-401-A93S) antibody was purchased from Rockland Immunochemicals. Anti-NS5A (9E10) antibody was kindly provided by Charles M. Rice^45^. Human IFN-α2a, IFN-λ1, and IFN-λ2 were purchased from PBL Assay Science. IFN-β was purchased from Pepro Tech. IFN-γ was purchased from Roche.

### Plasmids

LentiCRISPR v2 was kindly provided by Feng Zhang (Broad Institute, Addgene plasmid # 52961)^46^. To produce lentiCRISPR-Blast, the puromycin resistance gene in lentiCRISPR v2 was replaced with a blasticidin resistance gene from lentiCas9-Blast (Addgene plasmid #52962) using NEBuilder HiFi DNA Assembly Master Mix (New England BioLabs). The sgRNA sequences inserted into lentiCRISPR-Blast were STAT1 #1, TCATGACCTCCTGTCACAGC; STAT1 #2, GAGGTCATGAAAACGGATGG, STAT2, CCATCATAGCCCTTAAATCC; STAT3 #1, ACTGCTGGTCAATCTCTCCC; STAT3 #2, AGATTGCCCGGATTGTGGCC; STAT6 #1, TCCTGAGAACCCTCGTCACC; STAT6 #2, CATCAACAACACTGTGCCCT; IRF9 #1, GAACTGTGCTGTCGCTTTGA; IRF9 #2, GGAGCAGTCCATTCAGACAT; and NT, GGGGTAGGCCTAATTACGGA. These sequences were designed using the CRISPR Design Tool (http://crispr.mit.edu) or derived from previous studies^46^,^47^.

### Gene knockout using the CRISPR/Cas9 system

To produce lentivirus, HEK293T cells were transfected with 5 μg of lentiCRISPR-Blast, 2.5 μg of pCMV-VSV-G, and 3.75 μg of psPAX2 in a 10 cm-dish using GeneJuice (Novagen). After cells were incubated for 18 h, the media were replaced with 5 ml of fresh media supplemented with 1% BSA. Lentivirus-containing culture supernatants were collected after 24 and 48 h and were filtered through a 0.45 μm-pore size filter (Merck Millipore). Huh-7.5 cells were infected with lentivirus in the presence of 8 μg/ml Polybrene for 18 h. After the media were replaced with fresh media, cells were incubated for 24 h. Infected cells were selected using 4.0 μg/ml blasticidin for 7 days. To obtain knockout clones, 500 infected cells were mixed with 4 × 10^5^ uninfected cells and plated in a 10 cm-dish. After cells were incubated for 3 days, blasticidin was added to a final concentration of 4.0 μg/ml. Media were replaced every 5 or 6 days during selection. Cell colonies were collected using cloning rings (Iwaki). The absence of targeted gene products was confirmed by immunoblotting. Immunobloting was performed as described previously^44^. To identify the mutations introduced by the CRISPR/Cas9 system, genomic DNA was extracted using a NucleoSpin Tissue kit (Macherey-Nagel). Regions surrounding sgRNA target sequences were amplified by PCR. PCR products were directly sequenced or cloned into pCR-Blunt II-TOPO (Life Technologies) or pcDNA3 prior to sequencing.

### Real-time PCR

Cells were washed twice with PBS. Total RNA was extracted using a High Pure RNA isolation kit (Roche) or an RNeasy Mini kit (Qiagen). One hundred ng of total RNA was reverse transcribed using a ReverTra Ace kit (Toyobo). Real-time PCR was performed using a SYBR FAST qPCR kit (KAPA Biosystems) and a StepOne Plus real-time PCR system (Life Technologies). The primers used were HCV forward, CTTCACGCAGAAAGCGTCTA; HCV reverse, CAAGCACCCTATCAGGCAGT; PKR forward, TGGAAAGCGAACAAGGAGTAAG; PKR reverse, CCATCCCGTAGGTCTGTGAA; MX1 forward, ACACATGCTGAACATCACAGCTT; MX1 reverse, ACACGGCACTCATGCTCCTAA; ubiquitin forward, TGACTACAACATCCAGAA; and ubiquitin reverse, ATCTTTGCCTTGACATTC. Ubiquitin mRNA was used for normalization. To quantify HCV RNA, the standard curve was constructed as described previously^44^.

### Microarray analysis

Cells were left untreated or treated with IFN-α or IFN-λ for 24 h before total RNA was extracted using an RNeasy Mini kit. RNA integrity was verified with an Agilent Bioanalyzer. Sense-strand DNA was generated from 200 ng of total RNA, fragmented, and labeled using a GeneChip WT Plus Reagent Kit (Affymetrix). Hybridization and scanning were performed as described previously^48^, except that GeneChip HuGene 2.0 ST Arrays (Affymetrix) were used. Hierarchical clustering (uncentered correlation) was performed using Subio Platform version 1.19 (Subio).

### Statistical analysis

Data were analyzed by unpaired Student’s *t* test.

## Additional Information

**Accession codes:** Microarray data have been deposited in Gene Expression Omnibus (GEO) with accession number GSE84693.

**How to cite this article**: Yamauchi, S. *et al*. STAT1 is essential for the inhibition of hepatitis C virus replication by interferon-λ but not by interferon-α. *Sci. Rep.* **6**, 38336; doi: 10.1038/srep38336 (2016).

**Publisher's note:** Springer Nature remains neutral with regard to jurisdictional claims in published maps and institutional affiliations.

## Supplementary Material

Supplementary Information

Supplementary Dataset

## Figures and Tables

**Table 1:** Microarray data of select ISGs.

Gene Symbol	NT		STAT1 KO #1		
	IFN-α	IFN-λ	‒	IFN-α	IFN-λ
IFI6	75.35	81.32	1.00	62.72	0.89
IFIT1	50.58	57.44	1.29	74.81	0.55
MX1	47.00	74.05	1.01	66.72	1.11
IFIH1	40.82	60.52	1.06	49.77	1.01
ISG15	25.29	33.85	1.32	26.89	1.16
OAS2	21.80	40.09	0.80	20.08	1.15
OAS3	18.15	25.91	1.01	23.36	0.99
DDX60	16.70	27.45	0.84	19.02	0.95
HERC6	11.68	18.88	0.92	16.91	0.70
LGALS3BP	11.51	12.49	1.22	3.38	1.15
SP110	11.15	18.18	1.45	18.17	1.29
SAMD9	10.92	23.09	0.89	37.06	1.09
IFITM4P	9.70	10.50	1.08	9.45	1.51
PARP9	8.77	10.75	0.97	12.19	0.97
PARP14	8.40	14.01	1.06	16.63	1.22
IFIT5	8.27	9.95	1.24	14.03	1.04
BST2	7.86	10.82	0.90	2.18	1.13
EIF2AK2	6.84	7.60	1.06	7.67	1.02
DDX58	6.84	10.11	1.11	12.65	1.01
UBE2L6	6.74	11.49	0.88	9.39	0.72
IRF9	5.94	5.96	0.74	4.81	0.82
HELZ2	5.05	5.49	1.12	7.25	1.07
APOL6	4.64	7.47	1.13	11.63	1.22
DTX3L	4.64	5.04	1.01	6.38	0.92
PLSCR1	4.38	5.89	0.96	3.35	0.95
HERC5	4.15	7.80	0.99	15.36	0.76
CMPK2	3.93	7.59	0.76	4.58	0.86
TRIM25	3.80	3.84	0.98	3.56	0.91
SP100	3.71	6.26	1.30	5.29	1.23
USP18	3.63	5.50	1.06	7.62	1.01

NT sgRNA-expressing Huh-7.5 cells and STAT1 knockout (KO) cells (clone #1) were treated with IFN-α (1,000 U/ml) or IFN-λ (1,000 U/ml) for 24 h. Microarray analysis was performed. Fold changes relative to untreated NT sgRNA-expressing cells were calculated and were averaged for genes represented by multiple probe sets. The top 30 genes that were up-regulated by IFN-α in NT sgRNA-expressing cells were selected.
