# Supplementary material for: STAT1 is essential for the inhibition of hepatitis C virus replication by interferon-λ but not by interferon-α
Source: Sci Rep. 2016 Dec 8;6:38336. doi: 10.1038/srep38336 (PMC5144079; doi:10.1038/srep38336)
Supplement: Supplementary Information [file srep38336-s1.pdf]

STAT1 is essential for the inhibition of hepatitis C virus replication by interferon-λ but not by interferon-α

Shota Yamauchi[1,2,+], Kenji Takeuchi[1,2], Kazuyasu Chihara[1,2], Chisato Honjoh[1,3], Yuji Kato[1,4], Hatsumi Yoshiki[1], Hak Hotta[5], and Kiyonao Sada[1,2]

[1]Department of Genome Science and Microbiology, Faculty of Medical Sciences, [2]Life Science Innovation Center, [3]Third Department of Internal Medicine, Faculty of Medical Sciences, and [4]Department of Otorhinolaryngology Head and Neck Surgery, Faculty of Medical Sciences, University of Fukui, Fukui 910-1193, Japan. [5]Division of Microbiology, Kobe University Graduate School of Medicine, Kobe 650-0017, Japan. [+]Present address: Department of Cell Biology, University of Miami Miller School of Medicine, Miami, Florida, USA. Correspondence and requests for materials should be addressed to S.Y. (email: syamauchi@med.miami.edu)

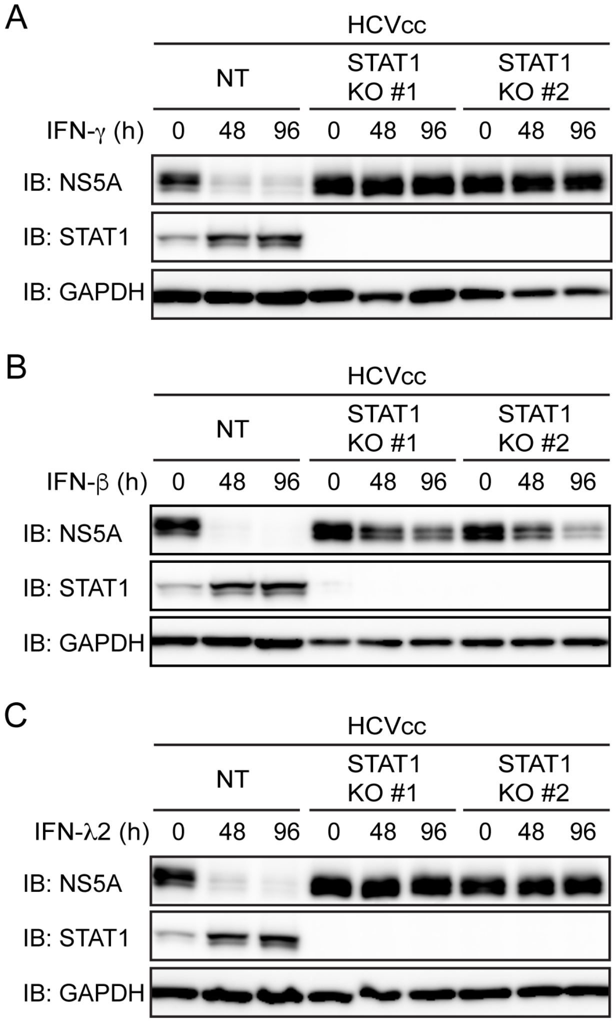

Supplementary Figure S1. Knockout of STAT1 abolishes the inhibition of HCV replication by IFN-λ2. (A-C) NT sgRNA-expressing cells and STAT1 KO cells (clones #1 and #2) were infected with HCVcc and treated with IFN-γ (1,000 U/ml) (A), IFN-β (1 ng/ml) (B), or IFN-λ2 (1,000 U/ml) (C) for the indicated times. The expression levels of NS5A and STAT1 were evaluated by immunoblotting (IB).

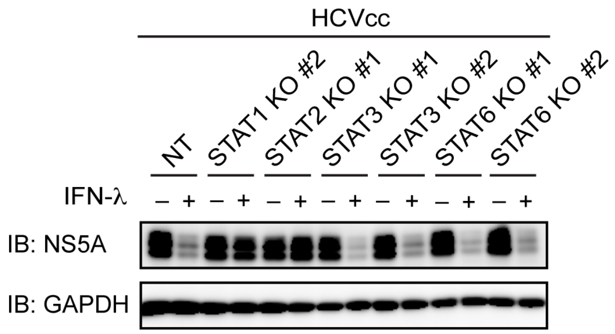

Supplementary Figure S2. Knockout of STAT3 or STAT6 does not affect the inhibition of HCV replication by IFN-λ. Cells were infected with HCVcc and treated with IFN-λ1 (1,000 U/ml) for 72 h. The expression level of NS5A was evaluated by immunoblotting (IB).
